# Investigating the Utility of Dopamine in Agricultural Practices: A Review

**DOI:** 10.3390/metabo15090586

**Published:** 2025-08-31

**Authors:** Wael M. Semida, Kareem Khalafallah Abdeltawab, Ashraf Sh. Osman, Mohamed H. H. Roby

**Affiliations:** 1 Horticulture Department, Faculty of Agriculture, Fayoum University, Fayoum 63514, Egypt; wms00@fayoum.edu.eg (W.M.S.); aso00@fayoum.edu.eg (A.S.O.); 2 Food Science and Technology Department, Faculty of Agriculture, Fayoum University, Fayoum 63514, Egypt

**Keywords:** agriculture, antioxidant, crop protection, dopamine, environmental stress, plant growth, sustainable farming

## Abstract

**Background/Objectives**: Dopamine (DA), a chemical commonly associated with neuroscience and human physiology, has been the subject of growing interest in the field of agriculture due to its potential applications. **Methods**: This comprehensive review examines the multifaceted role of dopamine in agricultural practices, elucidating its chemical characteristics, biological activities, and diverse applications. The review examines the chemical properties and physiological functions of dopamine in plants, highlighting the unique characteristics that make it suitable for agricultural applications. A significant portion of the review is dedicated to analyzing the biological activities of dopamine, particularly its antioxidant properties, and exploring the underlying mechanisms. The review also delves into the potential of dopamine to enhance crop growth, yield, and quality and investigates the influence of dopamine on plant physiology and metabolism. **Results**: Furthermore, the review provides a forward-looking perspective on the prospects of dopamine in agriculture, identifying emerging trends and areas of innovation that hold promise for sustainable and resilient farming systems. **Conclusions**: In summary, this review consolidates the current knowledge surrounding dopamine’s potential in agriculture, underscoring its versatility as a natural tool for growth enhancement and environmental sustainability, and offering valuable insights for researchers, practitioners, and policymakers seeking innovative approaches to address the challenges of modern agriculture.

## 1. Introduction

The agricultural sector faces a growing array of challenges, including biotic and abiotic stressors [1,2,3]. Researchers have increasingly explored the non-neuronal roles of neurotransmitters in both animal and plant systems [4]. The interest in the non-neuronal roles of neurotransmitters in plant systems emerged from the detection of these chemicals in substantial quantities within plant tissues. One compound that has captivated plant researchers is dopamine, a widely recognized animal neurotransmitter that is part of the biogenic amine group known as catecholamines; compelling evidence indicates that dopamine plays a vital role in plant physiology and development [5].

Dopamine (3,4-dihydroxyphenethylamine) is classified as a catecholamine compound with the molecular formula C_8_H_11_NO_2_. As the simplest possible catecholamine, dopamine belongs to a family that also includes the neurotransmitters norepinephrine and epinephrine. Structurally, dopamine consists of a benzene ring with two hydroxyl groups attached at the 3 and 4 positions (forming the catechol moiety) and an ethylamine side chain, which makes it a substituted phenethylamine [6].

This comprehensive review examines the multifaceted utility of dopamine in agricultural contexts, providing insights into its chemical pathway in plants, its characteristics, and varied practices. As the global agricultural sector grapples with growing complexities, such as biotic and abiotic stressors, climate variability, and the imperative for sustainable farming methods, dopamine presents a promising solution to these challenges [7,8,9,10]. Dopamine’s distinctive chemical properties and biological functions render it a compelling subject for investigation within the context of contemporary agriculture. This review explores the potential of dopamine in agriculture, illuminating its sources, biological functions, and applications for enhancing crop growth and productivity. Furthermore, we examine the environmental ramifications and obstacles linked to its utilization, offering a well-balanced viewpoint on its function in promoting environmentally sustainable agricultural practices. The subsequent sections will explore the complex body of knowledge surrounding the agricultural applications of dopamine, with the ultimate goal of providing valuable insights for researchers, practitioners, and policymakers who are seeking innovative approaches to advance the field of agriculture. To systematically explore the multifaceted utility of dopamine in agriculture, this review is structured to build from fundamental principles to practical applications. We begin by providing a foundational overview of dopamine, including its chemical properties and its established role as an antioxidant in plants. We then delve into the specific mechanisms of dopamine biosynthesis and regulation within plant systems, establishing the biological context for its function. Building on this, the review examines the broad physiological effects of dopamine on plant growth and development. The core of the review then focuses on its direct agricultural applications, presenting a detailed analysis of dopamine’s proven efficacy in enhancing plant tolerance to key abiotic stresses such as salinity, drought, and nutrient deficiency. Finally, we synthesize these findings to offer a forward-looking perspective, highlighting emerging trends and future research directions that promise to solidify dopamine’s role as a valuable tool for developing sustainable and resilient agricultural systems.

## 2. Overview of Dopamine Effects and Its Role in Plans

When plants face challenging environmental conditions, they demonstrate remarkable resilience through the secretion of specialized chemical compounds that help them survive abiotic stress. Among these protective molecules, dopamine stands out as a particularly fascinating example of nature’s adaptive mechanisms. As a member of the catecholamine family—a group of bio-amines characterized by their catechol-derived structures—dopamine shares its classification with epinephrine and norepinephrine. While these compounds are well-recognized in animals for their roles as hormones and neurotransmitters [11], their presence and function in plants reveal an intriguing evolutionary parallel.

The discovery that plants naturally synthesize several neurotransmitters found in animals, including both serotonin and dopamine, has opened new avenues of research in plant biochemistry [12]. Initially, dopamine’s role in plants was recognized primarily for its antioxidant properties, where it demonstrated superior protective capabilities compared to other well-known antioxidants such as catechin, glutathione, and flavonoids in counteracting oxidative damage [5]. This powerful free radical scavenging ability proves essential for maintaining cellular homeostasis, particularly in regulating ion transport between cells and supporting the critical process of chloroplast photophosphorylation [13].

Research has consistently demonstrated that dopamine significantly enhances plant stress resistance and environmental adaptability, making it a crucial component of plant defense systems. Beyond its antioxidant functions, dopamine plays a fundamental role in maintaining cell wall integrity and providing essential pathogen resistance mechanisms [14]. Remarkably, dopamine exhibits antioxidant capacity comparable to ascorbic acid and gallocatechin gallate, while surpassing the protective effects of quercetin, glutathione, luteolin, and catechin [15].

Under adverse environmental conditions, plants employ a remarkable survival mechanism—they secrete chemical compounds, such as dopamine, into their external environment, enabling them to endure abiotic stress. Catecholamines are a group of bio-amines with structures derived from catechol. Dopamine, epinephrine, and norepinephrine are examples of catecholamines. Catecholamines in animals serve as hormones and neurotransmitters due to their functions [11]. Plants also naturally contain several of the same neurotransmitters found in animals, including serotonin and dopamine [12]. Dopamine was first recognized as an antioxidant in plants, demonstrating a greater ability than catechin, glutathione, and flavonoids to counteract oxidative damage [5]. Dopamine possesses a potent ability to reduce free radicals, aiding in maintaining the equilibrium of reactive oxygen species. In general, dopamine enhances plant stress resistance and adaptability to unfavorable environments, as numerous research studies demonstrate. Dopamine is crucial for cell wall structure and essential for resisting pathogens [14].

## 3. Dopamine Biosynthesis and Regulation in Plants

The past several decades have witnessed a surge of scientific interest in understanding the non-neuronal functions of neurotransmitters across both animal and plant systems. This research momentum has been largely driven by the surprising discovery of these compounds in substantial concentrations within plant tissues. Dopamine, as a well-established animal neurotransmitter belonging to the catecholamine group of biogenic amines, has particularly captured the attention of plant scientists. The structural unity among catecholamines, including norepinephrine, epinephrine, and their derivatives, lies in their shared 3,4-dihydroxy phenyl ring configuration [16].

**Figure 1:**
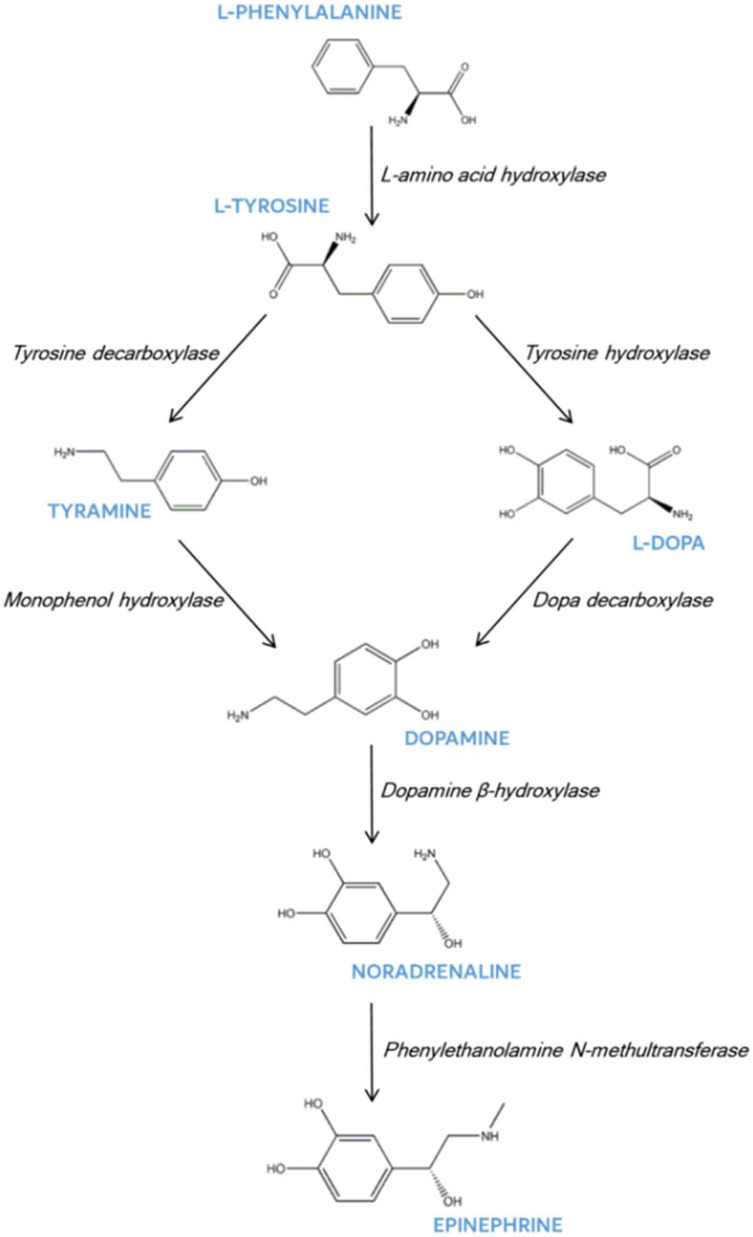
Dopamine biosynthetic pathways in plants [26].

In plants, dopamine biosynthesis follows pathways that mirror those found in animal systems, utilizing the amino acid tyrosine as the foundational precursor (Figure 1). This synthesis can proceed through two distinct routes: one pathway utilizes tyramine as an intermediate, while the alternative route proceeds through L-DOPA. Interestingly, the environmental conditions significantly influence this process, with dark-grown plant callus cultures showing elevated catecholamine biosynthesis and accumulation compared to their light-grown counterparts. However, it is important to note that only a select number of plant species naturally produce dopamine in significant quantities, and the specific cellular locations of dopamine production vary considerably among different species [5].

The regulatory mechanisms governing catecholamine function in plants appear to parallel their animal counterparts, potentially helping to modulate various metabolic processes, including the crucial scavenging of reactive oxygen species [17]. These compounds function as metabolic intermediates in both anabolic and catabolic cellular processes, with their physiological effects being concentration dependent. While moderate levels of catecholamines promote beneficial cellular activities, excessive concentrations can trigger undesirable physiological responses [18].

The enzymatic machinery involved in dopamine metabolism demonstrates the sophisticated nature of plant biochemical systems. When plants are supplied with exogenous DOPA, the enzyme DOPA decarboxylase efficiently converts it to dopamine, which then serves as a precursor for alkaloid biosynthesis, including important compounds such as morphine, thebaine, codeine, papaverine, and narcotine. Additionally, DOPA supplementation has been shown to enhance photosynthetic rates by promoting chlorophyll synthesis [19,20,21]. The oxidative pathway of dopamine involves tyrosinase-catalyzed conversion to dopamine quinone, which subsequently undergoes self-polymerization to form melanin. This process can be enhanced by the presence of phenolic compounds such as hydroxycinnamic acids [22].

The biosynthetic pathways for catecholamine production in plants demonstrate remarkable conservation with mammalian systems. Both pathways originate from tyrosine but diverge in their enzymatic steps [23,24]. The first route involves tyrosine-decarboxylase-mediated decarboxylation of tyrosine to produce tyramine, which is subsequently hydroxylated by monophenol hydroxylase to yield dopamine. The second pathway begins with the tyrosine-hydroxylase-catalyzed hydroxylation of tyrosine to generate levodopa, followed by dopa-decarboxylase-mediated decarboxylation to produce dopamine [24,25].

## 4. Physiological Effects of Dopamine in Plants

Plants synthesize dopamine from tyrosine via L-DOPA or via tyramine routes, accumulate it in variable amounts across species, and modulate its levels in response to developmental cues and environmental stress. Interest in plant dopamine has surged because exogenous and endogenous dopamine influence growth, redox balance, stomatal behavior, nutrient uptake, and stress tolerance, while sometimes producing negative effects depending on dose and context.

Dopamine exerts a significant influence on plant growth and development by interacting with phytohormones. Furthermore, dopamine plays a crucial role in the intracellular regulation of ion permeability and photophosphorylation within chloroplasts due to its reducing properties, ultimately leading to the scavenging of free radicals [5]. Dopamine plays a crucial role in regulating diverse physiological processes in plants, such as the metabolism of indole acetic acid, photosynthesis, flowering, defense against herbivory, and nitrogen fixation [27].

Plants possess enzymes that convert tyrosine to L-DOPA and then to dopamine (DOPA decarboxylase pathways), as well as an alternative route via tyrosine decarboxylation to tyramine followed by hydroxylation. The abundance of dopamine and its precursors varies widely among species and tissues (e.g., high in banana pulp), and biosynthetic gene expression can increase under drought, salinity, and pathogen attack. Dopamine can be methylated or oxidized; oxidative polymerization (melanin-like products) and amine oxidase activity are important routes for its catabolism. These metabolic features underpin dopamine’s dual character as both an antioxidant and pro-oxidant in plants.

Recent studies indicate that exogenous dopamine can preserve photosynthetic pigments and enhance photosynthetic efficiency under abiotic stress. For instance, in apple seedlings (*Malus domestica*), pretreatment with 100 µM dopamine significantly inhibited degradation of photosynthetic pigments and elevated net photosynthetic rate under drought stress. Moreover, dopamine reduced H_2_O_2_ accumulation while boosting antioxidant enzyme activity [28]. Under salinity stress, dopamine (100–200 µM) improved the net photosynthetic rate, photochemical efficiency (Fv/Fm), and chlorophyll content in *Malus hupehensis*, in addition to modulating ion homeostasis and ROS via enhanced antioxidative defense systems [29].

Dopamine acts as a potent antioxidant and modulates the plant’s oxidative response. In cucumber seedlings exposed to bisphenol A (BPA) stress, exogenous dopamine enhanced chlorophyll levels, growth, biomass accumulation, and root viability, while reducing ROS and MDA levels via elevated antioxidant enzyme activities and upregulated glutathione S-transferase expression [30].

In spinach and other species, dopamine maintains photosynthesis under stress by modulating stomatal behavior and enhancing CO_2_ utilization. This helps retain a high chlorophyll concentration and supports gas exchange, especially when nutrients are limited [26]. Under nutrient-deficient conditions, dopamine enhances photosynthetic rates by regulating stomatal conductance and retaining chlorophyll. It also promotes nutrient uptake and internal distribution by influencing root architecture and transport processes—with apples under potassium-deficient conditions displaying root modifications facilitating improved absorption Specifically, in apple seedlings under low-NH_4_^+^ conditions, dopamine improved root development, chlorophyll content, nitrogen uptake, and nitrogen metabolism, while also elevating antioxidant enzyme activity and the expression of nitrogen transporter genes [28].

Dopamine’s beneficial physiological effects are underpinned by extensive transcriptomic modulation. In apple, dopamine treatment under drought stress induced 1052 differentially expressed genes, including those related to nitrogen metabolism, secondary metabolite pathways, Ca^2+^ signaling, and transcription factors (WRKY, ERF, NAC), all of which contributed to improved stress tolerance [28].

## 5. Growth and Developmental Processes

The function of dopamine in plant physiology remains largely unexplored, although existing studies suggest it plays a role in multiple facets of plant growth and maturation [26]. Many studies have shown that catecholamines can interact with plant hormones, affecting how plants grow and develop. One example is dopamine, a type of catecholamine that can help plants produce more chlorophyll and maintain higher photosynthesis rates, especially when they are under stress, such as exposure to harmful hydrocarbons [10]. Increased dopamine levels enhance the auxin concentration by inhibiting indole acetic acid oxidase, thereby preventing the oxidation of IAA (indole-3-acetic acid), both in experimental settings and in living organisms. Given that dopamine negatively impacted root growth in soybean seedlings, two potential mechanisms were proposed. The inhibition may have been caused by the oxidation of dopamine, leading to the generation of reactive oxygen species, quinones, and semiquinones. Alternatively, the inhibition could have resulted from increased IAA levels due to dopamine’s inhibitory effect on IAA oxidase [10]. Moreover, exogenous dopamine stimulated ethylene biosynthesis in illuminated chloroplast lamellae extracted from sugar beet leaves [31]. High levels of hydrogen peroxide (H_2_O_2_) were found to reduce the chlorophyll content in detached tomato leaves, accelerating chlorophyll degradation under senescence conditions (10 mM H_2_O_2_ treatment for 8 h) [32] The application of exogenous DA decreased the synthesis of H_2_O_2_, resulting in higher chlorophyll levels and improved photosynthesis rates in plants affected by stress. In tomato (*Solanum lycopersicum*) seedlings exposed to salt stress (100 mM NaCl), exogenous dopamine (100 µM) application significantly reduced H_2_O_2_ and malondialdehyde (MDA) levels, while boosting the content of chlorophyll a, chlorophyll b, and total chlorophyll, and net photosynthetic activity. This finding is supported by a recent study demonstrating that under salinity stress conditions, dopamine treatment led to a ~31.9% reduction in H_2_O_2_, a 43–65% increase in chlorophyll content, and improvements in photosynthetic indicators [32]. In another study by [33], in apple seedlings (*Malus hupehensis*) subjected to alkali stress (pH 9.0 hydroponic solution), exogenous dopamine (0.1 mM) suppressed the accumulation of H_2_O_2_ by approximately 28.9%, preserved chlorophyll levels, and enhanced net photosynthesis and chlorophyll fluorescence (Fv/Fm). These observations are grounded in a study that detailed the antioxidative and photosynthetic improvements conferred by dopamine under alkaline stress conditions. Therefore, external application of DA is suggested to enhance chlorophyll production and photosynthetic activity in stressed plants by reducing oxidative damage [30].

## 6. The Agricultural Applications of Dopamine

Dopamine has demonstrated the potential to enhance plant biomass and regulate stress responses [10,34,35,36,37,38,39], thereby opening up a variety of promising applications (Table 1).

**Table 1:** Selected reviews of recent studies investigating the use of dopamine to improve plant growth and enhance resistance to biotic and abiotic stresses.

Method and Rate of Dopamine Application	Study Objectives	Outcomes	Reference
Foliar application of dopamine 0, 50, 100, 150, and 200 µM in watermelon	(Chilling stress)—Morphological, biochemical, and physiological	Significantly alleviates chilling stress. ↑ Growth, total chlorophylls, total carotenoids, *Pn*, antioxidant enzymes (SOD, CAT, POD), proline. ↑ Stimulating the activity of the PA metabolism. ↓ Lowering the accumulation of ROS, MDA, and H_2_O_2_.	[37]
Seed treatment of 100 and 200 µM dopamine in soybean	(Salt stress)—Morphological, biochemical, and physiological	↓ Reduced the impacts of salinity stress. ↑ Germination, growth, total chlorophylls, Fv/Fm, K^+^, N^+3^, and Ca^+2^ ions. ↓ Carotenoids, GB, TAA, TSP and Proline, Na^+^ ions. ↑ Synthesis of new DNA bands and the loss of bands formed under salinity stress.	[34]
Irrigation with 50, 100, and 200 µM dopamine in tomato seedlings	(Salt, and drought stress)—Morphological, biochemical, and physiological	↓ MDA, slightly reduced proline, superoxide, and H_2_O_2_. ↑ Plant dry weights, root length, FeSOD, and CAT2 gene expressions.	[35]
Foliar application of 0, 50, 100, and 200 µM dopamine in tomato seedlings	(Salt stress)—Morphological, biochemical, and physiological	↑ Growth, LRWC, SPAD, chl-a, chl-b, total chlorophyll, IAA content, K^+^/Na^+^ ratio, and Ca^2+^/Na^+^ ratio. ↓ MP, H_2_O_2_, MDA, proline, and sucrose contents, and CAT, POD, and SOD activities, ABA content.	[36]
Foliar application of dopamine concentrations (0, 50, 100 µM) on crisphead lettuce	(Nitrogen deficiency)—Morphological, biochemical, and physiological	↑ Growth, chlorophyll a, chlorophyll b, total chlorophyll, N, POD, CAT, and SOD, total sugar, total dissolved solids (TDS), vitamin C. ↓ ND, nitrate accumulation, H_2_O_2_, MDA, and O_2_^•−^, ROS.	[38]
Foliar application of dopamine concentrations (0, 50, 100, 150, 200 µM) on cucumber	(Nitrate stress)—Morphological, biochemical, and physiological	↑ Growth, chlorophyll b, carotenoid, Fv/Fm, qP, ETR, gas exchange, carbon metabolism. ↓ qN, nitrate-nitrogen concentration.	[10]

*Pn*: leaf net photosynthetic rate, SOD: superoxide dismutase, CAT: catalase, POD: peroxidase, MDA: malondialdehyde, H_2_O_2_: hydrogen peroxide, O_2_^•–^ superoxide radical, ROS: reactive oxygen species, PA: polyamines, Fv/Fm: photosynthetic activity, GB: glycine betaine, TAA: total amino acids, TSP: total soluble proteins, LRWC: leaf relative water content, SPAD: chlorophyll reading value, chl-a: chlorophyll a, chl-b: chlorophyll b, IAA: Indole acetic acid, MP: membrane permeability, ABA: abscisic acid, ND: nitrogen deficiency, qP: photochemical quenching, ETR: electron transport rate, qN: non-photochemical quenching.

Dopamine can enhance plant tolerance to abiotic stress such as drought, high salinity, and nutrient deprivation [8,40]. Wounding triggers a stress-response signaling cascade in plants, and dopamine is one of the molecules involved in modulating these responses. As an antioxidant and signaling compound, dopamine helps mitigate the oxidative damage generated during wounding and contributes to the activation of defense pathways, including those related to cell wall reinforcement and pathogen resistance. Therefore, the discussion of wounding responses is relevant because it provides context for understanding dopamine’s role as part of the broader plant defense network. The cascade of signaling molecules typically resembles the one activated in response to other types of stress. Additionally, the endogenous levels of certain hormones, such as jasmonic acid and auxins, also change in response to stress. Furthermore, increased dopamine concentrations have been observed in the wounded leaves of potato plants [41]. Stress is known to induce a temporary surge in reactive oxygen species. The significance of dopamine in redox signaling has increasingly drawn the focus of investigators. Dopamine’s antioxidant capabilities allow organisms to regulate their stress reactions [5]. *Solanum tuberosum* plants exhibit elevated dopamine concentrations when exposed to diverse abiotic stress, including drought, ultraviolet radiation, and abscisic acid treatments. Furthermore, the biosynthetic enzyme tyrosine decarboxylase displays enhanced activity under saline conditions [42]. Application of exogenous dopamine mitigated the detrimental effects of various abiotic stressors, such as drought, salinity, nutrient deficiency, alkalinity, cadmium toxicity, and waterlogging, in apple plants [43].

### 6.1. Salinity Stress

Hormones such as dopamine, melatonin, serotonin, etc., can be employed to mitigate the detrimental impacts of salinity stress on plant growth and development [44,45]. Salt stress results in the release of L-DOPA into the medium without inducing dopamine formation. The study by Li and others [29] investigated the effects of exogenous dopamine treatments on the physiological, morphological, and biochemical characteristics of tomato seedlings under saline conditions. A 100 mM NaCl solution was used to create salinity stress, and dopamine solutions were applied at 7-day intervals. Salt stress significantly inhibited plant growth, but dopamine treatments alleviated these negative effects. A 100 µM dopamine treatment enhanced plant and root dry weights, stem diameter, height, and leaf area compared to the control. Under salinity stress, parameters like LRWC, SPAD, chlorophyll a and b, and total chlorophyll decreased, while membrane permeability, H_2_O_2_, MDA, proline, sucrose, and enzyme activities increased. The application of 100 µM dopamine under salt stress improved these parameters compared to the control. When 200 µM dopamine was applied, stress-related compounds and enzyme activities decreased compared to non-dopamine-treated plants. Exogenous dopamine also increased IAA, decreased ABA, and improved K^+^/Na^+^ and Ca^2+^/Na^+^ ratios under salt stress. In conclusion, dopamine treatments effectively mitigated cellular damage and enhanced salt tolerance in tomato seedlings [36]. The study by Yildirim and others found that soybean seeds exposed to 150 mM NaCl had reduced germination, tolerance, and vigor. Salinity also decreased photosynthetic efficiency and chlorophyll content. Dopamine treatment helped mitigate the toxic effects of salinity, maintain ion balance, and enhance molecular responses [34].

### 6.2. Drought Stress

Dopamine has been shown to positively affect metabolic, physiological, and biochemical functions, as well as plant growth, under various environmental stressors [30]. Plants are better equipped to withstand abiotic stress thanks to modifications made at the cellular and molecular levels. This method typically uses a lot of energy, which stops plants from growing and producing as much. Stress may occasionally cause a 50–80% decrease in agricultural productivity [46].

In tomato seedlings, applying dopamine (along with progesterone) helped plants to cope with drought by boosting antioxidant enzyme activity and lowering ethylene production. This protected cell membranes, encouraged root growth, and highlights dopamine’s potential as a treatment to improve drought tolerance in tomatoes [35]. Dopamine has been observed to mitigate the deleterious effects of stress on photosynthetic processes and enhance water use efficiency and antioxidant defense [9,10,40].

### 6.3. Nutrient Stress

Recent studies have demonstrated that exogenous administration of 100 µM DA could bolster the crisphead lettuce plant’s tolerance to nitrogen deficiency by reducing ROS buildup and stimulating enzymatic antioxidants, concomitantly enhancing growth, yield, and quality [38]. In *Malus hupehensis*, dopamine application was observed to mitigate the detrimental effects of nutrient deficiency, alleviating the inhibition of growth, chlorophyll concentration, and net photosynthesis [40]. Under low-nitrogen conditions, plant growth was hindered, resulting in notable reductions in net photosynthetic rates, chlorophyll levels, and maximal quantum yield of PSII (Fv/Fm). Adding 100 μmol L^−1^ exogenous dopamine significantly reduced the effects of low-nitrogen stress on plant growth. Exogenous dopamine modified the uptake, conveyance, and dispersion of nitrogen, phosphorus, and potassium, while altering the root system structure in conditions with low nitrogen availability. External dopamine increases resistance to low-nitrogen stress by improving the function of enzymes involved in nitrogen metabolism, including nitrate reductase, nitrite reductase, glutamic acid synthase, and glutamine synthetase. Low-nitrogen conditions hinder plant growth, reducing photosynthetic rates, chlorophyll content, and PSII quantum yield. Application of 100 μmol L^−1^ exogenous dopamine alleviated these effects by enhancing nitrogen uptake, transport, and distribution, while also increasing the activity of key nitrogen-metabolizing enzymes [4].

## 7. Dopamine and Environmental Pollutants

This section synthesizes the evidence on how exogenous dopamine (DA) mitigates plant injury caused by environmental pollutants including bisphenol A (BPA), petroleum hydrocarbons (PHCs), and heavy metals. These stresses typically elevate reactive oxygen species (ROS), disrupt photosynthetic pigments, and impair growth; DA often counters these effects by enhancing antioxidant capacity, maintaining chlorophyll, and stabilizing photosynthesis.

Higher levels of soil contamination with crude oil result in abiotic stress for plants. Additionally, the heavy metals in hydrocarbons stimulate the production of reactive oxygen (ROS) species within plants. This oxidative stress can damage plant cells and impair their growth and development [45]. However, studies have shown that dopamine can help mitigate the effects of abiotic stress on plants [46]. Plants can employ various strategies to mitigate abiotic stress. They can manage hydrocarbon toxicity by utilizing cellular detoxification and controlling osmotic and ionic balance, largely through their anti-oxidative mechanisms [47]. While Zorb and others focused on salt stress, hydrocarbon toxicity presents a different but equally significant abiotic stress factor. Crude oil and petroleum-derived hydrocarbons can infiltrate soils, coating root surfaces and physically blocking water and nutrient absorption. Additionally, hydrocarbons often contain or mobilize heavy metals, which can enter plant tissues and disrupt metabolic pathways. These compounds stimulate excessive production of reactive oxygen species (ROS), causing oxidative stress that damages membranes, proteins, and nucleic acids. Hydrocarbon contamination can also alter the soil structure, reduce aeration, and alter the microbial community, further impairing nutrient cycling. In this context, dopamine’s antioxidant properties, membrane-protective effects, and role in sustaining ion transport are crucial in helping plants survive and function in response to hydrocarbon-induced toxicity [7]. Hydrocarbon-related pressure has emerged as a significant constraint on crop selection and yield in many regions across the globe.

In cucumber, exogenous DA alleviated BPA-induced phytotoxicity by lowering ROS (H_2_O_2_, superoxide) and lipid peroxidation (MDA), while boosting the activities/levels of antioxidant defenses and detoxification enzymes. Growth, biomass, and root viability improved concurrently, demonstrating a functional rescue of photosynthetic performance and cellular redox homeostasis [30]. While some studies report context-dependent or concentration-dependent DA effects (including potential growth inhibition in *Arabidopsis* at certain regimes), the pollutant-stress studies mentioned above consistently document protective outcomes at physiologically relevant DA doses. This nuance is acknowledged in our discussion of limitations [28].

## 8. Conclusions

Modern agriculture is subject to an expanding array of obstacles, including environmental stress and hydrocarbon pollution. Researchers have become increasingly interested in studying the non-neuronal functions of neurotransmitters in both animals and plants. This interest arose from the discovery of these chemicals in significant amounts within plant tissues. One such substance that has intrigued plant scientists is dopamine, a well-known animal neurotransmitter that belongs to the catecholamine biogenic amine group. Dopamine was first recognized as an antioxidant in plants that had a greater capacity than certain other antioxidant compounds to mitigate oxidative damage. Dopamine has shown promise in agricultural applications. Studies have demonstrated its ability to protect crop yields from abiotic and biotic stress. It can enhance crop growth, yield, and quality, offering hope for improved agricultural productivity. Dopamine’s versatility allows it to be used in diverse farming systems, from organic to controlled-environment agriculture, where it plays a crucial role in safeguarding crops and promoting growth. The evidence indicates dopamine is not only a growth-promoting agent but also a shield against agricultural adversity. As the agricultural sector seeks to balance productivity and sustainability, dopamine offers a timely solution. It has the potential to reduce reliance on synthetic chemicals and enhance food safety. By mitigating oxidative stress, preserving soil health, and contributing to sustainable practices, dopamine holds promise for a more resilient and environmentally conscious future in farming. While the evidence presented in this review consolidates dopamine’s potential, the transition from laboratory findings to widespread field application requires a focused research agenda to address critical knowledge gaps. First, future mechanistic studies should prioritize the identification and characterization of putative plant-specific dopamine receptors and downstream signaling components. Unraveling this molecular machinery is fundamental to understanding how an external signal is transduced into a physiological stress response. Second, the intricate crosstalk between dopamine and other phytohormone pathways, beyond auxin, remains largely uncharted. Comprehensive studies are needed to map these interactions to predict synergistic or antagonistic effects. Third, there is a pressing need for agronomic optimization. Research must move towards establishing optimal application doses, timings, and methods (e.g., foliar spray, seed priming, soil drench) for key commodity crops under variable field conditions. Finally, exploring the genetic basis of endogenous dopamine synthesis in high-producing species could pave the way for novel breeding strategies and biotechnological approaches aimed at developing crops with inherent stress resilience.

## Data Availability

No new data were created or analyzed in this study.

## References

[1] Semida W.M., Abdelkhalik A., Rady M.O.A., Marey R.A., El-mageed T.A.A. (2020). Exogenously applied proline enhances growth and productivity of drought stressed onion by improving photosynthetic efficiency, water use efficiency and up-regulating osmoprotectants. Sci. Hortic..

[2] Semida W.M., Abdelkhalik A., Mohamed G.F., Abd El-Mageed T.A., Abd El-Mageed S.A., Rady M.M., Ali E.F. (2021). Foliar Application of Zinc Oxide Nanoparticles Promotes Drought Stress Tolerance in Eggplant (*Solanum melongena* L.). Plants.

[3] Abdelkhalik A., Gyushi M.A.H., Howladar S.M., Kutby A.M., Asiri N.A., Baeshen A.A., Nahari A.M., Alsamadany H., Semida W.M. (2025). Synergistic Effects of Zinc Oxide Nanoparticles and Moringa Leaf Extracts on Drought Tolerance and Productivity of *Cucurbita pepo* L. Under Saline Conditions. Plants.

[4] Liu X.M., Gao T.T., Zhang Z.J., Tan K.X., Jin Y.B., Zhao Y.J., Ma F.W., Li C. (2020). The mitigation effects of exogenous dopamine on low nitrogen stress in Malus hupehensis. J. Integr. Agric..

[5] Kulma A., Szopa J. (2007). Catecholamines are active compounds in plants. Plant Sci..

[6] Jerzemowska G. (2016). Hypothalamic and Midbrain Cells, Tyrosine Hydroxylase, and Implications for Drug Addiction. Neuropathology of Drug Addictions and Substance Misuse Volume 3: General Processes and Mechanisms, Prescription Medications, Caffeine and Areca, Polydrug Misuse, Emerging Addictions and Non-Drug Addictions.

[7] Ahmad A., Khan W.U., Shah A.A., Yasin N.A., Ali A., Rizwan M., Ali S. (2021). Dopamine Alleviates Hydrocarbon Stress in Brassica oleracea through Modulation of Physio-Biochemical Attributes and Antioxidant Defense Systems. Chemosphere.

[8] Liang B., Gao T., Zhao Q., Ma C., Chen Q., Wei Z., Li C., Li C., Ma F. (2018). Effects of exogenous dopamine on the uptake, transport, and resorption of apple ionome under moderate drought. Front. Plant Sci..

[9] Gao T., Wang Y., Liu Y., Ma M., Li X., Zhang D., Ding K., Li C., Zou Y., Ma F. (2021). Overexpression of tyrosine decarboxylase (MdTYDC) enhances drought tolerance in Malus domestica. Sci. Hortic..

[10] Lan G., Jiao C., Wang G., Sun Y., Sun Y. (2020). Effects of dopamine on growth, carbon metabolism, and nitrogen metabolism in cucumber under nitrate stress. Sci. Hortic..

[11] Kuklin A.I., Conger B.V. (1995). Catecholamines in Plants. J. Plant Growth Regul..

[12] Bamel K. (2020). Prabhavathi Dopamine in Plant Development and Redox Signaling. Neurotransmitters in Plant Signaling and Communication.

[13] Kanazawa K., Sakakibara H. (2000). High content of dopamine, a strong antioxidant, in Cavendish banana. J. Agric. Food Chem..

[14] Facchini P.J., Yu M., Penzes-Yost C. (1999). Decreased Cell Wall Digestibility in Canola Transformed with Chimeric Tyrosine Decarboxylase Genes from Opium Poppy 1. Am. Soc. Plant Physiol..

[15] Jung S., Kim J.S., Cho Y., Tae G.S., Kang B.G. (2000). Antioxidant responses of cucumber (*Cucumis satius*) to photoinhibition and oxidative stress induced by norflurazon under high and low PPFDs. Plant Sci..

[16] Wang S., Che T., Levit A., Shoichet B.K., Wacker D., Roth B.L. (2018). Structure of the D2 dopamine receptor bound to the atypical antipsychotic drug risperidone. Nature.

[17] Leng Q., Mercier R.W., Yao W., Berkowitz G.A. (1999). Cloning and First Functional Characterization of a Plant Cyclic Nucleotide-Gated Cation Channel 1. Plant Physiol..

[18] Applewhite P.B., Bcxsdanski D.F., Pletscher A., Brodie B.B., Udenfriend S., Pbarm Exper I., Clark G., FENsrER E.D., Towne J.C. (1973). Serotonin and Norepinephrine in Plant Tissues. Phytochemistry.

[19] Camargo S.M.R., Vuille-Dit-Bille R.N., Mariotta L., Ramadan T., Huggel K., Singer D., Götze O., Verrey F. (2014). The molecular mechanism of intestinal levodopa absorption and its possible implications for the treatment of Parkinson’s disease. J. Pharmacol. Exp. Ther..

[20] Khan F., Qidwai T., Shukla R.K., Gupta V. (2013). Alkaloids derived from tyrosine: Modified benzyltetrahydroisoquinoline alkaloids. Natural Products: Phytochemistry, Botany and Metabolism of Alkaloids, Phenolics and Terpenes.

[21] Sourkes T.L. (1971). Actions of Levodopa and Dopamine in the Central Nervous System. JAMA.

[22] Rosei M.A., Blarzino C., Foppoli C., Mosca L., Coccia R. (1994). Lipoxygenase-catalyzed oxidation of catecholamines. Biochem. Biophys. Res. Commun..

[23] Steiner U., Schliemann W., Strack D. (1996). Assay for tyrosine hydroxylation activity of tyrosinase from betalain-forming plants and cell cultures. Anal. Biochem..

[24] Kong K., Lee J., Park H., Cho S. (1998). Purification and characterization of the tyrosinase isozymes of pine needles. IUBMB Life.

[25] Lundström J. (1971). Biosynthesis of mescaline and tetrahydroisoquinoline alkaloids in *Lophophora williamsii* (Lem.) Coult. Occurrence and biosynthesis of catecholamine and other intermediates. Acta Chem. Scand..

[26] Liu Q., Gao T., Liu W., Liu Y., Zhao Y., Liu Y., Li W., Ding K., Ma F., Li C. (2020). Functions of dopamine in plants: A review. Plant Signal. Behav..

[27] Roshchina V.V. (2022). Biogenic amines in plant cell at norma and stress: Probes for dopamine and histamine. Emerging Plant Growth Regulators in Agriculture.

[28] Gao T., Zhang Z., Liu X., Wu Q., Chen Q., Liu Q., van Nocker S., Ma F., Li C. (2020). Physiological and transcriptome analyses of the effects of exogenous dopamine on drought tolerance in apple. Plant Physiol. Biochem..

[29] Li C., Sun X., Chang C., Jia D., Wei Z., Li C., Ma F. (2015). Dopamine alleviates salt-induced stress in *Malus hupehensis*. Physiol. Plant..

[30] Ahammed G.J., Wang Y., Mao Q., Wu M., Yan Y., Ren J., Wang X., Liu A., Chen S. (2020). Dopamine alleviates bisphenol A-induced phytotoxicity by enhancing antioxidant and detoxification potential in cucumber. Environ. Pollut..

[31] Shull T.E., Kurepa J., Smalle J.A. (2023). Dopamine Inhibits Arabidopsis Growth through Increased Oxidative Stress and Auxin Activity. Stresses.

[32] Abo-Shanab W.A., Diab R.H. (2024). Dopamine Hydrochloride Alleviates the Salt-induced Stress in *Glycine max* (L.) Merr. plant. J. Soil Sci. Plant Nutr..

[33] Akcay U.C., Gencay R., Koc F.Z. (2024). Effect of dopamine and progesterone on the physiological and molecular responses of tomato seedlings to drought and salt stress. Cogent Food Agric..

[34] Yildirim E., Ekinci M., Turan M., Yuce M., Ors S., Araz O., Torun U., Argin S. (2024). Exogenous dopamine mitigates the effects of salinity stress in tomato seedlings by alleviating the oxidative stress and regulating phytohormones. Acta Physiol. Plant..

[35] Jiao C., Lan G., Sun Y., Wang G., Sun Y. (2021). Dopamine Alleviates Chilling Stress in Watermelon Seedlings via Modulation of Proline Content, Antioxidant Enzyme Activity, and Polyamine Metabolism. J. Plant Growth Regul..

[36] Farouk S., El-Hady M.A.M.A., El-Sherpiny M.A., Hassan M.M., Alamer K.H., Al-Robai S.A., Ali E.F., El-Bauome H.A. (2023). Effect of Dopamine on Growth, Some Biochemical Attributes, and the Yield of Crisphead Lettuce under Nitrogen Deficiency. Horticulturae.

[37] Abdulmajeed A.M., Alharbi B.M., Alharby H.F., Abualresh A.M., Badawy G.A., Semida W.M., Rady M.M. (2022). Simultaneous Action of Silymarin and Dopamine Enhances Defense Mechanisms Related to Antioxidants, Polyamine Metabolic Enzymes, and Tolerance to Cadmium Stress in Phaseolus vulgaris. Plants.

[38] Liang B.W., Li C.Y., Ma C.Q., Wei Z.W., Wang Q., Huang D. (2017). Dopamine alleviates nutrient deficiency-induced stress in Malus hupehensis. Plant Physiol. Biochem..

[39] Szopa J., Wilczyn´ski G.W., Fiehn O., Wenczel A., Willmitzer L. (2001). Identification and quantification of catecholamines in potato plants (*Solanum tuberosum*) by GC-MS. Phytochemistry.

[40] Świȩdrych A., Lorenc-Kukuła K., Skirycz A., Szopa J. (2004). The catecholamine biosynthesis route in potato is affected by stress. Plant Physiol. Biochem..

[41] Cao Y., Du P., Yin B., Zhou S., Li Z., Zhang X., Xu J., Liang B. (2023). Melatonin and dopamine enhance waterlogging tolerance by modulating ROS scavenging, nitrogen uptake, and the rhizosphere microbial community in Malus hupehensis. Plant Soil.

[42] Liu H., Fu Y., Hu D., Yu J., Liu H. (2018). Effect of green, yellow and purple radiation on biomass, photosynthesis, morphology and soluble sugar content of leafy lettuce via spectral wavebands “knock out”. Sci. Hortic..

[43] Abd El-mageed S.A., Sayed A.A.S., Shaaban A., Hemida K.A., Abdelkhalik A., Semida W.M., Mohamed I.A.A., Gyushi M.A., Elmohsen Y.H.A., Abd El Mageed T.A. (2025). Integrative application of licorice root extract and melatonin improves faba bean growth and production in Cd-contaminated saline soil. BMC Plant Biol..

[44] Das A.J., Kumar R. (2016). Bioremediation of petroleum contaminated soil to combat toxicity on Withania somnifera through seed priming with biosurfactant producing plant growth promoting rhizobacteria. J. Environ. Manag..

[45] Odukoya J., Lambert R., Sakrabani R. (2019). Understanding the impacts of crude oil and its induced abiotic stresses on agrifood production: A review. Horticulturae.

[46] Ahammed G.J., Li X. (2023). Dopamine-induced abiotic stress tolerance in horticultural plants. Sci. Hortic..

[47] Zörb C., Schmitt S., Neeb A., Karl S., Linder M., Schubert S. (2004). The biochemical reaction of maize (*Zea mays* L.) to salt stress is characterized by a mitigation of symptoms and not by a specific adaptation. Plant Sci..

